# Chromatin Remodeler Smarca5 Is Required for Cancer-Related Processes of Primary Cell Fitness and Immortalization

**DOI:** 10.3390/cells11050808

**Published:** 2022-02-25

**Authors:** Shefali Thakur, Vincent Cahais, Tereza Turkova, Tomas Zikmund, Claire Renard, Tomáš Stopka, Michael Korenjak, Jiri Zavadil

**Affiliations:** 1 Epigenomics and Mechanisms Branch, International Agency for Research on Cancer, World Health Organization, 69008 Lyon, France; thakurs@iarc.fr (S.T.); cahaisv@iarc.fr (V.C.); renardc@iarc.fr (C.R.); 2 Faculty of Science, Charles University, 128 43 Prague, Czech Republic; thakurs@iarc.fr (S.T.); 3 Biocev, First Faculty of Medicine, Charles University, 252 50 Vestec, Czech Republic; tereza.turkova@lf1.cuni.cz (T.T.); tomas.zikmund@helmholtz-muenchen.de (T.Z.); tomas.stopka@lf1.cuni.cz (T.S.); 4 Institute of Epigenetics and Stem Cells (IES), Helmholtz Zentrum, D-81377 München, Germany; tomas.zikmund@helmholtz-muenchen.de (T.Z.)

**Keywords:** Smarca5, Snf2h, cell immortalization, cell cycle, homologous recombination, non-homologous end-joining, senescence, ATAC-seq, RNA-seq, MEF

## Abstract

Smarca5, an ATPase of the ISWI class of chromatin remodelers, is a key regulator of chromatin structure, cell cycle and DNA repair. Smarca5 is deregulated in leukemia and breast, lung and gastric cancers. However, its role in oncogenesis is not well understood. Chromatin remodelers often play dosage-dependent roles in cancer. We therefore investigated the epigenomic and phenotypic impact of controlled stepwise attenuation of Smarca5 function in the context of primary cell transformation, a process relevant to tumor formation. Upon conditional single- or double-allele *Smarca5* deletion, the cells underwent both accelerated growth arrest and senescence entry and displayed gradually increased sensitivity to genotoxic insults. These phenotypic characteristics were explained by specific remodeling of the chromatin structure and the transcriptome in primary cells prior to the immortalization onset. These molecular programs implicated Smarca5 requirement in DNA damage repair, telomere maintenance, cell cycle progression and in restricting apoptosis and cellular senescence. Consistent with the molecular programs, we demonstrate for the first time that Smarca5-deficient primary cells exhibit dramatically decreased capacity to bypass senescence and immortalize, an indispensable step during cell transformation and cancer development. Thus, Smarca5 plays a crucial role in key homeostatic processes and sustains cancer-promoting molecular programs and cellular phenotypes.

## 1. Introduction

Chromatin remodeling is a quintessential step for all DNA-associated core cellular processes, many of which determine key cell fate decisions, including those implicated in cancer onset and evolution [1]. The role of chromatin remodeling in cell transformation and cancer development has been of increasing interest over the last decade [2,3,4,5], and various chromatin remodelers have been intimately linked to carcinogenesis [6,7,8,9,10,11,12]. Findings from large-scale epigenomics and transcriptomics studies highlight the frequent mutation or deregulation of chromatin remodelers in cancer [8,13], suggesting that aberrant chromatin remodeling plays an important role in cancer development. Four major classes of chromatin remodelers are described [14], each containing a conserved core Snf2 helicase [15] that uses the energy from ATP hydrolysis to disrupt the contacts between histones and DNA [16,17]. One such ATPase, belonging to the highly conserved ISWI family of chromatin remodelers, is Smarca5 [18]. It plays an important role in nucleosome assembly and spacing and contributes to processes involved in cell fate decisions, including DNA replication [19], repair [20,21,22] and transcription via all three classes of RNA polymerase [23].

*Smarca5* is frequently overexpressed in different cancer types, including glioma [24], leukemia [25], breast [26,27] and gastric [28] cancer. In contrast, Smarca5 loss in cancer cell lines (by depletion using siRNA) [29] and in murine hematopoietic, progenitor cells [30] as well as murine oocytes [31] using complete knockout has been shown to negatively impact cell proliferation [29,30]. *Smarca5* deletion is embryonically lethal in mice and the derived embryonic cells [32]. Loss of Smarca5 also impacts sensitivity to radiation exposure [33] and gene expression of DNA repair genes [24,30]. It also causes chromosomal aberrations [34], indicative of genomic instability [35]. Hence, Smarca5 has a major impact on pathways involved in cell transformation, cancer development and evolution. However, these changes have not been investigated and reported in terms of Smarca5 dosage variation and may provide important insights into its function. This is particularly relevant because chromatin remodelers often have a dosage-sensitive role in the genesis of human cancer [8]. To address this, we assessed for the first time the effects of gradual Smarca5 decrease in a model of primary cell immortalization based on primary mouse embryonic fibroblasts (MEFs) using single and double allele knockout. The rationale for using this model is two-tiered. Firstly, the consequences of Smarca5 knockout or knockdown are frequently studied in transformed cell lines [29,36], which are not ideal for studying the early stages of cell transformation. In addition, available knowledge on Smarca5 molecular targets and functions in primary cells comes from embryonic stem cells [37,38] in the context of development and not oncogenic cell transformation. Primary MEFs are a validated model for investigating early steps of cell immortalization and transformation via cellular senescence bypass, during which they closely recapitulate molecular programs and events observed in human cancers [39,40,41,42,43]. Secondly, due to the pronounced effects of complete Smarca5 loss on phenotype, it is challenging to dissect phenotypic and molecular alterations caused by Smarca5 loss from changes observed due to compromised cellular fitness in the double-knockout cells. Thus, focusing only on the molecular and phenotypic changes common to both single and double allele knockout cells can overcome this issue. Using massively parallel sequencing, we studied the effects of gradual loss of Smarca5 on global chromatin accessibility and gene expression programs, two key processes controlled by Smarca5 [23,44,45]. Smarca5 loss in the primary cells affected gene regulation programs involving DNA damage repair, DNA replication, telomere maintenance, chromosomal segregation, apoptosis and cell aging. Complementing the gene regulation analyses, functional studies of the cell phenotype outcomes upon Smarca5 loss revealed increased susceptibility of the primary cells to chemical mutagens and severely decreased cell immortalization potential. By integrating comprehensive analyses at the molecular as well as phenotypic levels, our study delineates the critical role of Smarca5 in primary cell fitness and the early stages of the cancer-like process of primary cell immortalization, characteristics relevant to cancer onset and evolution.

## 2. Materials and Methods

### 2.1. Genetically Modified Mice

*Smarca5^fl^* allele contains two loxP1 sites flanking the exon 5 of the *Smarca5* gene. Cre mediated recombination of *Smarca5^fl^* allele causes removal of the region critical for enzymatic activity of Smarca5 as well as produces a frameshift mutation resulting in disruption of Smarca5 protein expression. *Smarca5^fl^* was produced in our laboratory as previously described [30]. The murine strain expressing a tamoxifen-inducible Cre Recombinase-Estrogen receptor (Cre-Esr1) fusion protein [B6.Cg-Tg(CAG-cre/Esr1*)5Amc/J, Stock No: 004682] was purchased from The Jackson Laboratory (Bar Harbor, ME, USA) [46]. The reporter R26-stop-EYFP strain [B6.129X1-Gt(ROSA)26Sortm1(EYFP)Cos/J, Stock No: 006148] was kindly provided by Dr. Vladimir Korinek. Mice were maintained in individually ventilated cages with unlimited supply of water and food as well as regular bedding exchange. All experiments met criteria approved by Czech ministry of agriculture and committee for experimental animals. For the genotyping of mice, we isolated genomic DNA from tail tips and performed PCR using Sapphire Amp Fast PCR Master Mix (RR350A, Takara Bio, Kusatsu, Shiga, Japan) with following primers: S5fl-fwd: 5′-ACTGAGGACTCTGATGCAAACAGTCAAG-3′, S5fl-rev: 5′-TACACAACTAAGGCAGTGGGTTATAGTGC-3′, S5del-fwd: 5′-GTGCAAAGCCCAGAGACGATGGTATG-3′ (with S5fl-rev, for identification Cre recombined S5fl allele), Cre-fwd: 5′-ACCAGGTTCGTTCACTCATGG-3′, Cre-rev: 5′-ACGGGCACTGTGTCCAGACC-3′, EYFP-fwd: 5′-AAGACCGCGAAGAGTTTGTC-3′, EYFP-rev: 5′- AAAGTCGCTCTGAGTTGTTAT-3′.

### 2.2. Mouse Embryonic Fibroblast Isolation and Maintenance

*Smarca5^fl^* mice were crossed with Cre-Esr1 and R26-stop-EYFP strains to obtain parental lineages with suitable genotypes for following breeding and MEF isolation. Pregnant female mice at 14th day post-coitum were sacrificed, washed with 70% ethanol and transferred into flow box. From each mouse, both uterine horns were isolated, quickly washed with 70% ethanol and placed into Petri dish with PBS. Embryos were separated from placenta and extra-embryonic tissues and then transferred into 12-well plate. Head and organs of each embryo were carefully removed and used for genomic DNA isolation and genotyping. The rest of the body was washed in PBS, finely minced using razor blade and incubated with 1 mL 0.05% trypsin/0.02%EDTA/DNAse I (100U) solution for 15–20 min at RT in 35 mm sterile dishes. After the embryonic tissue was dissociated, the resulting cell suspension was resuspended in MEF culture medium (DMEM containing 4.5 g/L glucose without phenol red supplemented with 10% FBS, 1% Penicillin-Streptomycin, 1% non-essential amino acids, 2 mM *L*-glutamine). The suspension from each embryo was individually plated to the 0.2% gelatin-coated 10-centimeter petri dish and incubated at 37 °C, 5% CO_2_. After the fibroblasts were attached to the surface of the culture dish (within approximately 30–60 min), they were washed twice with warm PBS and cultured in MEF medium in incubator until they reached 80% confluence. All fibroblast cultures were then frozen in MEF medium containing 20% FBS and 10% DMSO and kept in liquid nitrogen until the genotyping results were known. Since Cre-Esr1 fusion construct is activated by presence of phenol red in culture media, the MEF cells were strictly maintained in media without this dye. 

### 2.3. Cell Culture

MEFs were grown in DMEM culture medium without phenol red (Sigma-Aldrich, St. Louis, MO, USA). Cell count and viability were measured using trypan blue staining and Bio-Rad TC20 automated cell counter. For conditional deletion of *Smarca5^fl^* allele, 4-Hydroxytamoxifen (4-OHT) (100 nM final, H6278, Sigma-Aldrich) was added for 6 h into the medium. Successful activation of the Cre-Esr1 fusion protein was verified by comparing EYFP positivity of 4-OHT treated and untreated cells using a CytoFLEX flow cytometer (Beckman Coulter, Brea, CA, USA). *Smarca5^fl^* allele deletion in the Cre-Esr1 activated cells was analyzed by PCR from genomic DNA using S5del-fwd and S5fl-rev primers. 

### 2.4. Immunoblotting

MEFs were collected, washed with PBS and lysed 30 min in lysis buffer (150 mM NaCl, 50 mM Tris-Cl pH 7.5, 0.4% Triton-X, 2 mM CaCl_2_, 2 mM MgCl_2_, 1 mM EDTA, 5 mM NaF in dH_2_O) supplemented with 1 mM DTT, protease (TPCK, TLCK, PMSF) and phosphatase inhibitors (Na_3_VO_4_) and 25U/µL non-specific DNA nuclease (Benzonase; SC-391121, Santa Cruz Bitoechnology; Dallas, TX, USA) on ice. After 30 min, 2% SDS solution was added into each lysate in 1:1 ratio (final conc. of SDS 1%) and tubes containing protein lysates were heated for 5 min at 95 °C. Protein lysates were then cleared by centrifugation at 16,000× *g*, 10 min, 4 °C and subjected to bicinchoninic acid assay (Thermo Fisher; #23228, Waltham, MA, USA) in order to determine total protein concentration. 20 µg of the protein was resolved on SDS gradient 4–15% Mini-PROTEAN TGX Precast Protein gels (Bio-Rad Laboratories, Basel, Switzerland) and semi-dry transferred by Trans-Blot Turbo Transfer System (Bio-Rad Laboratories) using manufacture’s settings to PVDF membrane (Bio-Rad Laboratories; #162-0177). Primary antibodies used were anti-Smarca5 (1:1000; Bethyl Laboratories; A301-017A, Montgomery, TX, USA), anti-p21 (1:1500; SC-6246; Santa Cruz Biotechnology, Dallas, TX, USA) and anti-GAPDH (1:2500; Sigma-Aldrich; HPA040067). After overnight incubation with primary antibody, membranes were washed 3 × 10 min with TBST and stained 1h with peroxidase-conjugated donkey Anti-Rabbit IgG secondary antibody (1:10,000; ab20662; Abcam, Cambridge, UK) for anti-Smarca5, donkey Anti-Rabbit IgG secondary antibody (1:10,000; 711-036-152; Jackson ImmunoResearch, Ely, UK) for anti-GAPDH and donkey Anti-Mouse IgG secondary antibody (1:10,000; 715-036-150; Jackson ImmunoResearch) for anti-p21 primary antibody. Protein signal was visualized using Pierce™ ECL Western Blotting Substrate (Thermo Fisher; #PI32106) and detected and quantified with the ChemiDoc Imaging System (Bio-Rad Laboratories).

### 2.5. Detection of Cellular Senescence

MEFs were plated in 35-millimeter well plates and cultured as described above. The senescence detection was carried out for each studied cKO genotype between 96 h and passages 7–9 when the cells exhibited typical senescent morphology (enlarged cell size, doubled nuclei), by using the Cellular Senescence Assay Kit (Merck; KAA002, Darmstadt, Germany) which detects the pH-dependent senescence-associated β-galactosidase (SA-β-gal) activity. The resulting blue cell staining was recorded by brightfield microscopy (Primovert, Zeiss, Jena, Germany)/digital photography. The standard manufacturer’s (Merck; KAA002, Darmstadt, Germany) protocol and instructions were used without any modifications.

### 2.6. ATAC-Sequencing and Data Analysis

The cells were harvested four days after induction with 4-OHT and ATAC was performed on 7.5 × 10^4^ cells, in replicate for each condition, using a previously established protocol [47]. Transposase enzyme and Illumina compatible primers and barcodes for multiplexing were used as described earlier [48]. Multiplexed libraries were sequenced as 75 bp paired-end reads on the Illumina NextSeq 500 sequencer. Reads in FASTQ files were analyzed for data amount and quality using FastQC (v0.11.9) and trimmed with Trim Galore (cutadapt 0.6.4_dev, https://github.com/FelixKrueger/TrimGalore (accessed on 24 September 2019)), then mapped on the mouse mm10 genome using the Burrows–Wheeler Aligner (0.7.15) [49]. Duplicate reads were flagged by samblaster (v0.1.24) [50], and the aligned reads further underwent base quality score recalibration and indel realignment with the corresponding tools from GATK (v3.8) [51]. The Nextflow pipeline used is available at https://github.com/IARCbioinfo/alignment-nf. An average of 44 million reads was sequenced per sample. Model-based analysis of ChIP-seq (MACS2) [52] was used to call peaks within each sample, and a reproducible set of peaks between duplicates was defined with IDR [53] (irreproducible discovery rate). These sets were used as input for the DiffBind R/bioconductor package (Differential binding analysis of ChIP-Seq peak data), in which they are merged and reduced to the same size (200 bp on each size from the summit). Reads are counted in each sample for each peak of this global peak set, and this count is normalized with DESeq2 (Differential gene expression analysis based on the negative binomial distribution) native normalization method (referred to as DBA_NORMALIZATION_RLE in DiffBind). 

ATAC-seq peaks were annotated according to their genomic location (500 bp, 2 kb or 10 kb upstream of transcription start site (TSS), within gene body and/or overlapping exons) based on GENECODE annotation [54], with closest gene name (from peak center to TSS), replication timing information [55] (https://www2.replicationdomain.com/index.php, accessed on 5 October 2021) and candidate cis-regulatory element information [56] (https://screen.encodeproject.org/, accessed on 3 February 2021) including histone mark and CTCF binding information. Peaks were assigned to genes as described by Iurlaro et al. [37], except only the capture Hi-C interactions [57] common to both embryonic stem cells and fetal liver cells were used, assuming that promoter interactions common to both the pluripotent and the committed fetal cells are non-cell-of-origin specific and thus likely to be more relevant to MEFs than those exclusive to either of them. Using this approach, we assigned about 60% of the identified ATAC-peaks to genes.

### 2.7. Motif Analysis

Fold-change was calculated for differentially accessible ATAC peaks by dividing the average normalized count for the single or double allele knockout by the average of the normalized count for the corresponding wildtype. The MOtif aNAlysis with Lisa (monaLisa) suite [58] (https://fmicompbio.github.io/monaLisa/articles/monaLisa.html, accessed on 14 January 2022) was used to group the genomic regions into five bins based on fold-change assigned to each region for the single and the double allele knockout individually. Motif enrichments were then calculated for each bin and stability selection-based regression approach was used to predict binding preferences of transcription factors or nucleic acid binding proteins using min.score cutoff (=10) for motif detection.

### 2.8. RNA-Sequencing and Data Analysis

Experiment was performed in duplicates. 1 × 10^6^ cells were resuspended in Trizol and RNA was extracted using phenol-chloroform extraction [59]. RNA quality was assessed using Agilent Tapestation 4200 and all samples exhibited RNA Integrity Number (RIN) of 9.5 or higher. RNA libraries were prepared using KAPA Stranded RNA-seq kit with KAPA RiboErase (HMR) kit (Roche, Basel, Switzerland) to deplete rRNA. Multiplexed samples were sequenced as 75 bp paired-end reads on the Illumina NextSeq 500 sequencer. Reads were trimmed and aligned against the mm10 genome build with STAR; then the read number per gene per sample was counted with htseq-count (using the Nextflow pipeline described in https://github.com/IARCbioinfo/RNAseq-nf, accessed on 18 February 2020). An average of 34 million reads was sequenced per sample. The read count was normalized using the normalization method based on median of ratios, i.e., counts divided by sample-specific size factors determined by median ratio of gene counts relative to geometric mean per gene, embedded in DESeq2 R/Bioconductor package [60].

### 2.9. Differential Analysis of ATAC Accessibility Domains and Gene Expression

**Figure 1:**
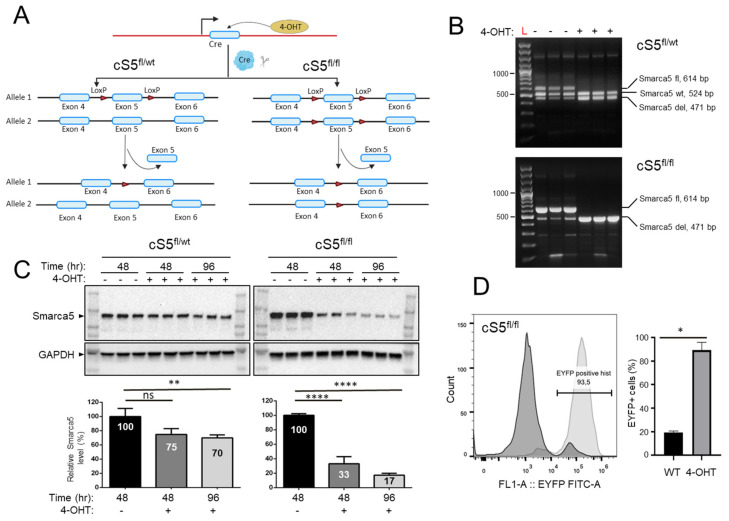
Characterization of the *Smarca5* deletion system. (**A**) Schematics of the *Smarca5* conditional knockout cassette. The cS5^fl/fl^ cells have the exon 5 of both *Smarca5* alleles floxed, while cS5^fl/wt^ MEFs have a single floxed *Smarca5* allele. The floxed allele(s) is excised upon 4-hydroxytamoxifen (4-OHT) addition. Some elements of the figure were created using BioRender.com. (**B**) Genotyping of the MEFs to confirm *Smarca5* deletion status using PCR. The wildtype *Smarca5* allele, the floxed allele and the allele with the exon 5 deletion differ in size and can thus be differentiated from each other, revealing the cell identity. DNA was isolated from cells 48 h after adding 4-OHT. Genotyping was done in biological triplicates, each line represents MEF cell line isolated from different embryos with CreER cS5^fl/fl^ or CreER cS5^fl/wt^ genotype. The low-representation bands below 300 bp are non-specific background amplicons. (**C**) Immunoblots (in the same triplicates used for genotyping) for anti-Smarca5 antibody reveal protein level depletion over time (48 h and 96 h) in the 4-OHT induced, *Smarca5* knockout MEFs compared to control without 4-OHT. A gradual protein level depletion is observed over time. (**D**) Confirmation of Cre-activation upon 4-OHT administration as assessed by expression of the EYFP, 96h after induction. Experiments were done in triplicates, MEF isolated from 3 embryos with CreER^+/−^ cS5^fl/fl^ EYFP^+/+^ genotype. Percent of cells expressing EYFP indicate percent of cells that are also positive for *Smarca5* knockout. Dark curve represents cells without 4-OHT and light curve represents same MEFs 96 h after 4-OHT induction, WT/4-OHT = untreated/treated with 4-OHT. ns = *p* > 0.05; * = *p* ≤ 0.05; ** = *p* ≤ 0.01; **** = *p* ≤ 0.0001.

The globally normalized value matrices for both ATAC-seq and RNA-seq data were further normalized by dividing the average values of the two Smarca5-deficient replicates by the average of their respective wild-type baseline values, followed by log_2_ transformation. The gradual differential changes from the single to the double allele knockout were identified using Pavlidis Template Matching (PTM) analysis tool [61] within the TMeV suite [62,63]. PTM allows statistically significant matching of the experimentally obtained data profiles to predefined profile templates. In the used PTM settings, the wild-type for each condition was treated as baseline with a value of 0.5 (a midpoint between the minimum of 0 and maximum of 1). For ATAC-seq, the gradual changes were probed based on a template following the Smarca5 genotype (allele) increments (100% to 50% to 0%) with a *p*-value of 0.025, i.e., for the downregulated genes, the single allele knockout was set at 0.25 and the double allele knockout was set at 0. Following this logic, for the upregulated peaks or genes, the single allele knockout was set at 0.75 and the double allele knockout was set at 1. The differential gene expression changes were probed based on both genotype and protein levels (100%–Wild-type to 69.8%–single allele knockout to 17.4%–double allele knockout; see Figure 1C) with a template matching *p*-value of 0.05. 

### 2.10. One-Way-ANOVA

A one-way ANOVA was also performed for the four replicate experimental groups (two knockout types and their corresponding wildtype samples) at *p*-value of 0.05 (alpha settings). The common events from PTM for the gradual upregulation based on genotype were subtracted from the resulting ANOVA-identified gene list. The same was done for downregulated genes based on genotype to obtain an ANOVA exclusive gene list comprising of residual reproducibly modulated genes that follow patterns not overlapping with those corresponding to the gradual increase or decrease along with the shift from the single-allele to the double-allele knockout.

### 2.11. Pathway and Network Analysis

A union of the genotype and protein-level based differential expression gene lists obtained by PTM was created. Upregulated and downregulated genes were individually analyzed by the functional annotation tool of DAVID v6.8 [64]. Deregulated and differentially regulated genes were probed for gene ontology (GO BP, GO MF, GO CC) and functional annotations with an EASE score of 0.1. Differentially regulated genes were overlapped with custom-built lists of cancer drivers or epigenetic regulator genes (ERGs) [13]. We curated the cancer driver gene list by combining information from Cancer Gene Census [65], IntOgen database [66] and Cancer drivers as defined by Bailey et al. [67]. The derived list was converted to orthologous mouse genes using Ensembl BioMart (https://www.ensembl.org/biomart/martview/, accessed on 20 April 2021). Network analysis using GeneMANIA [68] was performed on the deregulated cancer drivers and ERGs using the tool’s default parameters. From the available GeneMANIA network categories [68], interactions based on co-expression, physical interaction (based on protein-protein interaction data), genetic interaction and co-localization were included. In addition, when sufficient interactions were not available for these mentioned network categories, predicted interactions were included.

### 2.12. MTS Assay

Cells were plated in 96-well plates in triplicates and exposed to a range of doses of aristolochic acid-I (AA) for 24 h and methylnitronitrosoguanidine (MNNG) for 2 h. Metabolic activity of the cultures, as a proxy for cell viability, was measured using CellTiter 96 AQueous One Solution Cell Proliferation Assay (Promega, Fitchburg, WI, USA). Plates were incubated for 2 h at 37 °C and absorbance was measured at 492 nm. The percentage of viable cells in the carcinogen-exposed cultures was expressed relative to that of unexposed control cells, which was set to 100%.

### 2.13. Senescence Bypass and Immortalization

Twenty-five independent MEF cultures (T75 flasks) for each of the 4 conditions, i.e., 4-OHT induced and the corresponding un-induced wild-type cells, were cultured until the cells underwent growth arrest and cellular senescence. Cell count and viability were measured using trypan blue staining and Bio-Rad TC20 automated cell counter. This period of cellular senescence typically lasts several weeks [40]. Senescence onset and bypass were assessed by plotting growth curves for each culture, as population doublings against number of days in culture [69]. This extensive experiment was repeated and validated twice more using ten and six independent MEF cultures for each condition, respectively. 

## 3. Results

### 3.1. Loss of Smarca5 Deregulates Global Chromatin Accessibility and Gene Expression

The *Smarca5* double allele knockout has profound phenotypic effects [30]; therefore, we developed single allele knockout MEFs to allow us to inspect dosage-specific molecular and phenotypic changes associated with gradual Smarca5 loss. Floxed *Smarca5* single (cS5^fl/wt^) and double allele (cS5^fl/fl^) MEFs (Figure 1A), in conjunction with conditional Cre-mediated recombination, were used to generate a *Smarca5* frame-shift null mutation [30] upon induction with 4-OHT. The same cells without the 4-OHT induction were used as corresponding wild-type controls. The *Smarca5* deletion status of the MEFs was confirmed by PCR and flow cytometry (Figure 1B,D and Supplementary Figure S1A). We assessed the Smarca5 protein levels by immunoblotting and observed about 17% of residual protein in double allele knockout cells in the bulk culture compared to the wild-type samples 96 h after deletion. The single allele knockout cells expressed about 70% of residual protein at the same time point (Figure 1C). Thus, the conditional knockout MEFs exhibited a gradual decrease in gene and protein dosage from wild type to single and single to double allele knockout cells. Since Smarca5 protein levels in the double allele knockout cells are largely diminished 96 h after induction of deletion, we investigated genome-wide molecular changes four days after the knockout (Supplementary Figure S1B). At this time point, the knockout cells remain mostly viable (>90% viability).

To study the effects of loss of chromatin remodeler Smarca5 on global chromatin accessibility, we used ATAC-seq, a technique that allows genome-wide identification of accessible sites using a hyperactive transposase [48]. RNA-seq was used in parallel to assess gene expression changes, another key process controlled by Smarca5 [45]. We first inspected the obtained ATAC-seq and RNA-seq data for quality and reproducibility between replicates (Supplementary Figure S1C,D) and then performed differential peak calling and expression analysis on the datasets. All described analyses were carried out specifically considering the gradually decreasing Smarca5 doses between single and double knockout cells, i.e., only those differential changes in chromatin accessibility and gene expression that manifested in the same direction (up or down) in both genotypes and reflected the gradual change of Smarca5 doses (smaller differential change in single allele knockout, larger differential change in double allele knockout) were considered for interpretation. This strategy not only minimized the inclusion of changes attributable to compromised cellular fitness observed in the double allele knockout MEFs but also allowed us to focus on molecular changes that are Smarca5 dosage-dependent. For RNA-seq, the directional analysis was carried out using either genotypic (Supplementary Tables S1 and S2) or protein-based dosage determined in Figure 1C (Supplementary Tables S3 and S4) (see Section 2). A one-way ANOVA was also performed to identify all statistically meaningful differential changes, from which the above-mentioned genotypic dosage-dependent changes were subtracted. This resulted in an additional 805 differentially expressed genes that are provided in the Supplementary Materials (Supplementary Table S5). These genes, however, had a distinct and varied profile behavior and were thus not considered and biologically interpreted in association with targeted probing of Smarca5 dosage-dependent molecular programs.

**Figure 2:**
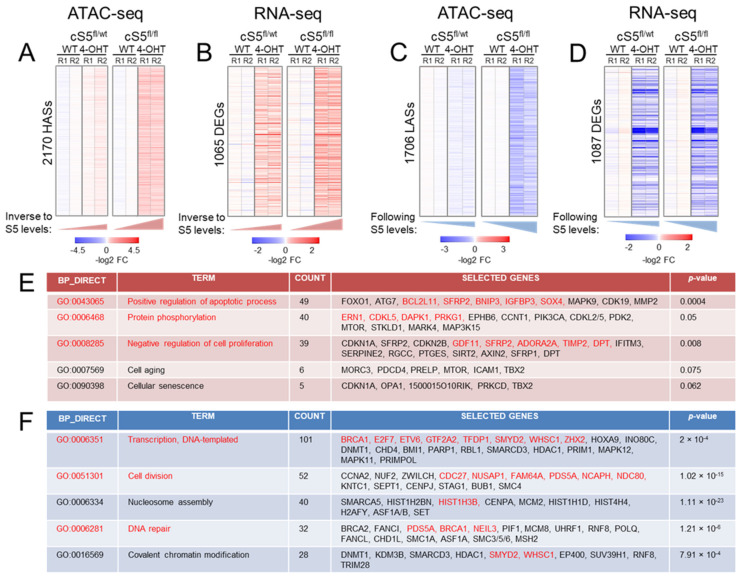
Epigenomic and transcriptomic remodeling upon the gradual loss of Smarca5. (**A**) Heatmap showing differentially accessible ATAC peaks displaying higher accessibility in the knockout cells, *p*-value–0.025. Notice the gradual increase in accessibility from the single allele knockout (cS5^fl/wt^, 4-OHT) to the double allele knockout (cS5^fl/fl^, 4-OHT) compared to their respective wildtypes (WT), HASs = higher accessibility sites. (**B**) Heatmap showing gradually upregulated genes from single to double-allele knockout MEFs, *p*-value–0.05. DEGs = differentially expressed genes. Notice the gradual increase in gene expression from cS5^fl/wt^, 4-OHT to the cS5^fl/fl^, 4-OHT compared to their respective wildtypes. (**C**) Heatmap showing differentially accessible ATAC peaks displaying lower accessibility in the knockout cells, *p*-value–0.025. Notice the gradual decrease in accessibility from the single allele knockout to the double allele knockout. LASs = lower accessibility regions. (**D**) Heatmap showing the gradually downregulated genes in the knockout cells, *p*-value–0.05. (**E**) Gene Ontology pathways (GO) in upregulated genes as interrogated by DAVID. Text in red shows events (pathways/genes) common in both chromatin accessibility and gene expression dataset as inspected by ATAC-seq and RNA-seq. (**F**) GO pathways in downregulated genes as interrogated by the DAVID tool (human orthologue gene symbols are shown as a default output of DAVID). Text in red font shows events (pathways/genes) common to both chromatin accessibility and gene expression datasets. WT = cells untreated with 4-OHT; log2 FC–log2-transformed fold-change from the WT baseline condition; R1, R2 = experimental replicates 1 and 2.

ATAC-seq analysis revealed thousands of regions following a dosage-specific pattern of either increased (Figure 2A, Supplementary Table S6) or decreased chromatin accessibility (Figure 2C and Supplementary Table S7). We noticed a slightly higher number of regions that gained accessibility (higher accessibility sites: HAS) compared to those with decreased accessibility (lower accessibility sites: LAS). Motif analysis of LASs with the highest fold-change revealed enrichment of, among others, the CTCF and FOS::JUN motifs in the single allele knockout cells (Supplementary Figure S2, Bin1). This is in keeping with the established role of Smarca5 in facilitating CTCF chromatin binding [45]. Interestingly, LASs with the highest fold-change in the double allele knockout show enrichment for only FOS::JUN binding motifs (Supplementary Figure S3, Bin1), indicative of increased deregulation of cell proliferation and stress response [70,71] with stepwise loss of Smarca5. Differential gene expression analysis on the replicates also revealed more than a thousand genes exhibiting gradual deregulation (up- or downregulation) (Figure 2B,D and Supplementary Tables S1–S4). These findings indicate massive chromatin and transcriptomic remodeling following the loss of Smarca5.

### 3.2. Loss of Smarca5 in Primary Cells Leads to Dosage-Specific Deregulation of Pathways Involved in Cell Proliferation and Genomic Stability

**Figure 3:**
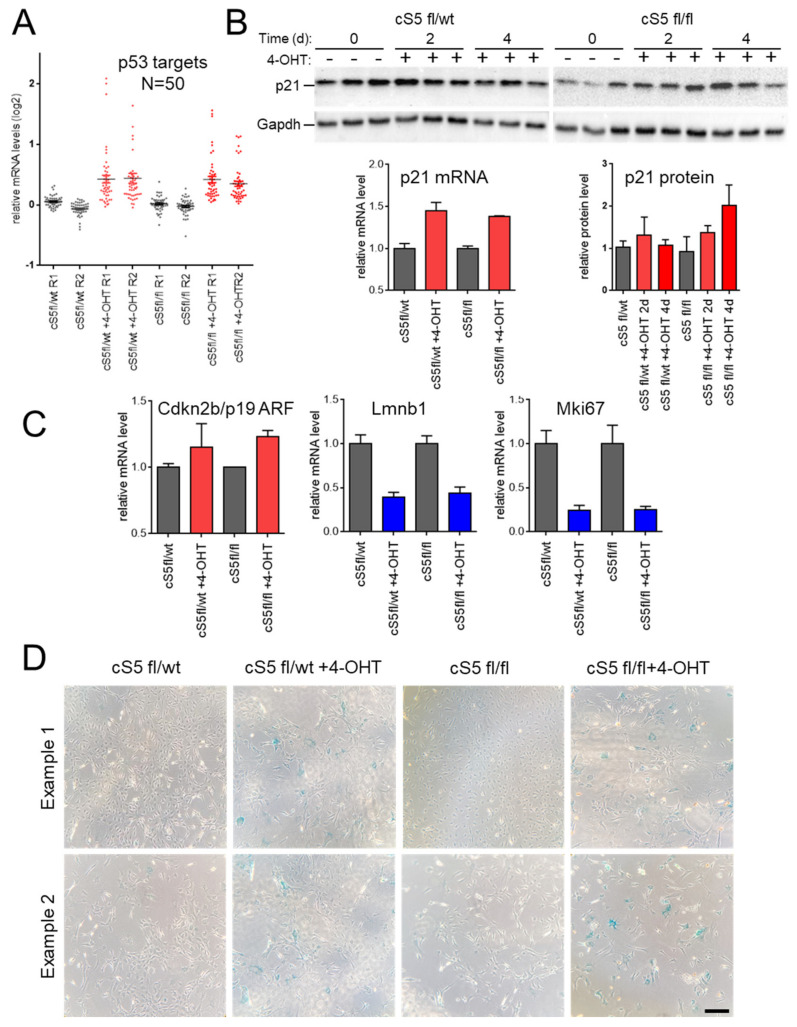
Effects of Smarca5 loss on senescence markers and phenotypes. (**A**) Induction of 50 direct p53 target genes upon treatment with 4-hydroxy-tamoxifen (4-OHT). (**B**) Immunoblotting and RNA-seq analyses of p21/Cdkn1a, a p53-dependent inducer of senescence. (**C**) RNA-seq analysis of senescence and cell-cycle exit markers. (**D**) Staining for senescence–associated SA-β-gal activity observed in the 4-OHT-treated cultures (already at 96 h post-treatment for *Smarca5* double-knockout (cS5^fl/fl^)). Scale bar = 100 μm.

To better understand the biological pathways affected by this major Smarca5 dosage-specific transcriptional reprogramming, a union of gradually deregulated genes based on the gene and protein dosage (see Section 2) was interrogated for Gene Ontology (GO) attributions. This analysis revealed marked enrichment of gradually downregulated genes, from single to double allele knockout, involved in various aspects of cell proliferation. We also observed enrichment for genes involved in the maintenance of genomic integrity, including the major DNA damage repair pathways (nucleotide excision repair, base excision repair, double-strand break repair via homologous recombination and non-homologous end-joining (Supplementary Table S8). Similarly, genes involved in nucleosome remodeling, chromatin organization and histone modification were affected. The datasets were also probed for KEGG and BIOCARTA pathways and we observed an enrichment of similar biological pathways (data not shown). To further support our findings, we interrogated the deregulated genes using EnrichR, the results of which were largely in agreement with the observations described above (data not shown). For upregulated genes, GO annotation revealed Smarca5 dosage-dependent enrichment of processes related to cell stress and cell death including autophagy, apoptosis and negative regulation of cell proliferation (Supplementary Table S9). This is in line with the enrichment of FOS:JUN motifs in LASs in the knockout cells (Supplementary Figures S2 and S3). In addition, upregulated genes were found involved in cytokine response and a number of signaling pathways, including Wnt, MAP kinase, mTOR, p53 and Ras signaling. A total of 50 p53 targets (Supplementary Table S10) [72] were induced in the knockout cells (Figure 3A), which further validates the cellular stress response in the absence of Smarca5. We also observed enrichment of cell aging/cellular senescence, including the Trp53-Cdkn1a/p21 pathway. This is consistent with the increased levels of p21 at the mRNA and protein levels (Figure 3B), deregulation of senescence and cell cycle exit markers (Figure 3C) and increased SA-β-gal activity (Figure 3D). 

We noticed that the expression of many genes involved in chromatin and epigenetic regulation was altered upon gradual Smarca5 loss. Therefore, we took advantage of a previously curated list of epigenetic regulator genes (ERGs) [13] that write, modify/revert and read epigenetic modifications in the cell to cross-reference them with the deregulated genes identified in expression analysis. Numerous ERGs were deregulated in primary MEFs upon *Smarca5* inactivation (Supplementary Table S10). Network analysis of the deregulated ERGs (Supplementary Figures S4 and S5), using GeneMANIA [68], confirmed their involvement in processes already identified by the GO analysis of all deregulated genes (e.g., DNA damage response–downregulated; cell aging–upregulated). More than 25 ERGs also exhibit differential chromatin accessibility changes upon knockout, including *Fbxo17*, *Smyd2* (Supplementary Figure S6).

Pathway analysis using genes that exhibit both differential accessibility and gene expression revealed a set of phenotypically relevant and commonly deregulated pathways between ATAC-seq and RNA-seq data (Figure 2E,F). If the analysis is extended beyond differential ATAC peaks found in gene promoters/proximal elements (Supplementary Table S11), using gene enhancer interactions based on available Hi-C data [57], as described in the methods, we find even more overlap between the two datasets. More than 180 differentially accessible chromatin regions can be linked to deregulated genes, with concerted changes in accessibility and gene expression (Supplementary Tables S12 and S13, Supplementary Figures S6 and S7). Thus, we report for the first time a catalogue of gradually deregulated genes and associated differentially accessible regulatory regions in the absence of Smarca5 that are linked to the disruption of major homeostatic pathways required for cellular fitness.

### 3.3. Stepwise Allelic Knockout of Smarca5 Results in Dosage-Specific Effects on Cellular Fitness

**Figure 4:**
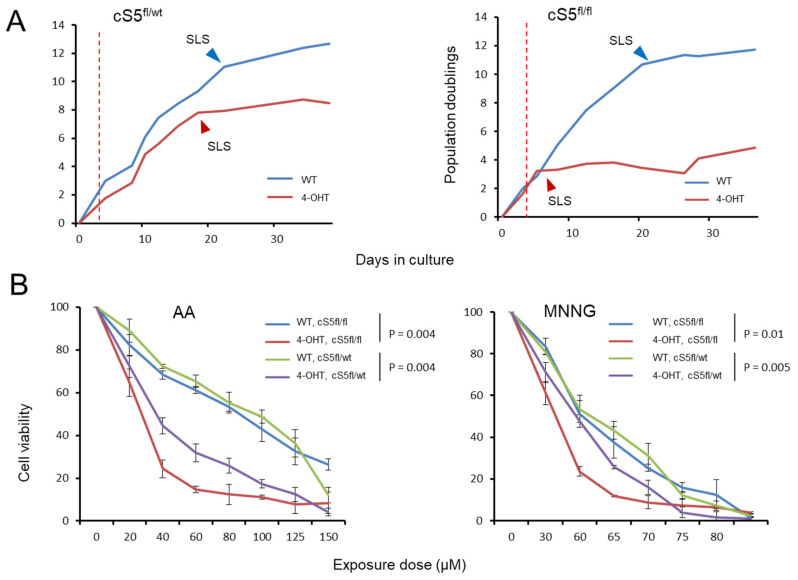
Impact of the stepwise *Smarca5* deletion on cell growth and cell viability upon genotoxic insults. (**A**) Growth curve for the *Smarca5* knockout MEFs when grown in culture for prolonged periods of time, (**left**): single allele knockout, (**right**): double allele knockout. Red dotted lines indicate the point of 4-OHT induction, arrowheads indicate the onset of senescence-like state (SLS). (**B**) Cell viability of single and double allele knockout *Smarca5* MEFs as measured by MTS assay upon exposure to different doses of AA in triplicates (**left**) and MNNG (**right**). NOTE: *p*-values in (**B**) are based on paired *t*-test for each genotype group.

Considering the above-described deregulated molecular programs, we characterized *Smarca5* single and double knockout primary MEFs with respect to their growth potential. For this, the cells were cultured for an extended period of 35–40 days and growth curves were generated (Figure 4A). The double knockout cells underwent growth arrest almost immediately after 4-OHT induction, entering a prolonged phase of a cellular senescence-like state (SLS), while the corresponding wild-type cells entered the SLS considerably later (Figure 4A, right panel). This SLS is characterized by an almost complete absence of cell growth, low cell viability and SA-β-gal activity (Figure 3D and Figure 4) and is typical for primary MEFs under stress conditions [40,73]. Analysis of the knockout cells revealed the accumulation of SA-β-gal staining in the double allele knockout already four days after *Smarca5* deletion, in morphologically still pre-senescent cells (Figure 3D), in keeping with the accelerated induction of SLS shown in Figure 4. The SLS was also characterized at the molecular level by inspecting levels of p21/Cdkn1a, a p53-dependent inducer of senescence (Figure 3B), and senescence markers like cell-cycle exit marker Mki67 and p19 ARF or lamin B1 (Figure 3C). We observed concomitant gene upregulation (Figure 3A) and increased accessibility of p53 targets like *Dapk1*, *Igfbp3* and *Bcl2l11* (Supplementary Figure S7) in the knockout cells, further validating this SLS in response to the Smarca5 loss. The single knockout MEFs displayed growth cessation and SLS onset ten to fourteen days after 4-OHT addition (Figure 4A, left panel), i.e., later than the double allele knockout MEFs but slightly earlier than their corresponding 4-OHT un-induced, wild-type cells These findings highlight dosage-dependent effects of Smarca5 on cell proliferation in extended growth assays, in line with the deregulated cell cycle, senescence and apoptosis programs identified by the molecular characterization of the cells.

In the past, Smarca5 loss has been linked to DNA repair defects [20,74] and has been studied in response to radiation damage [21,36,74,75]. In support of this, we found several DNA damage pathways gradually deregulated in the primary MEF knockout cells. Thus, we decided to study the impact of Smarca5 dosage on the cells’ sensitivity to genotoxic insult as a substitute functional readout for their deregulation of DNA damage repair. After deleting *Smarca5* allele(s), we exposed the cells to strong mutagens, namely AA and MNNG, a potent environmental/iatrogenic mutagen and a research mutagenic compound, respectively. AA induces ample TA to AT transversions via specific adduct formation while MNNG, an alkylating agent, causes high rates of CG to TA transitions [41] The double knockout cells showed the highest level of sensitivity to either exposure, displaying the most prominent decrease in cell viability (Figure 4B). The single knockout cells also showed higher sensitivity to mutagen exposure than the corresponding wild-type culture, but the effect was less pronounced than in the double knockout cells (Figure 4B). To exclude the possibility that the observed differences in cell viability are the result of underlying (genotypic) differences between the single and the double allele knockout MEFs since they are derived from different crosses, we directly compared the wild-type MEFs of the two genotypes side-by-side, for all exposure doses (Supplementary Figure S8). We did not observe major differences between the two wild types (Supplementary Figure S8A,B, left panels). However, we did observe differences between the two knockout conditions (Supplementary Figure S8A,B, right panels). This suggests that the differences in cell viability between the single and the double allele knockout MEFs are likely due to changes in Smarca5 levels rather than the baseline genetic make-up of the cells. In summary, characterization of the single and double allele knockout MEFs revealed Smarca5 dosage-dependent effects on cell growth, SLS onset and the sensitivity of the cells to mutagen exposure. 

### 3.4. Smarca5 Loss Impairs the Capacity of Primary Cells to Immortalize

Since *Smarca5* is overexpressed in different human cancers [24,25,26,28], we next asked whether known cancer driver genes were deregulated in the obtained gene expression and chromatin accessibility data. The cancer driver genes used for this analysis were based on an in-house curated list from a set of seminal studies [65,66,67] as described in the methods section. More than fifty cancer driver genes showed gradually altered chromatin accessibility upon single and double-allele *Smarca5* knockout and more than 100 driver genes exhibited differential gene expression (Supplementary Table S10). Some cancer drivers displayed both decreased accessibility and decreased mRNA levels, e.g., *Brca1*, *E2f7* and *Myh11* (Supplementary Figure S6), while others displayed increased accessibility and associated mRNA upregulation, e.g., *Msi2*, *Bace2* (Supplementary Figure S7). Network analysis of these deregulated cancer drivers revealed a general trend for the downregulated genes to be involved in processes important for cellular and genetic stability, such as DNA damage repair, telomere maintenance and chromosome segregation (Supplementary Table S8 and Supplementary Figure S9). The upregulated cancer drivers, in line with the observed Smarca5 dosage-dependent phenotypic outcomes, were principally involved in processes including apoptosis and response to cellular stress (Supplementary Table S9 and Supplementary Figure S10).

**Figure 5:**
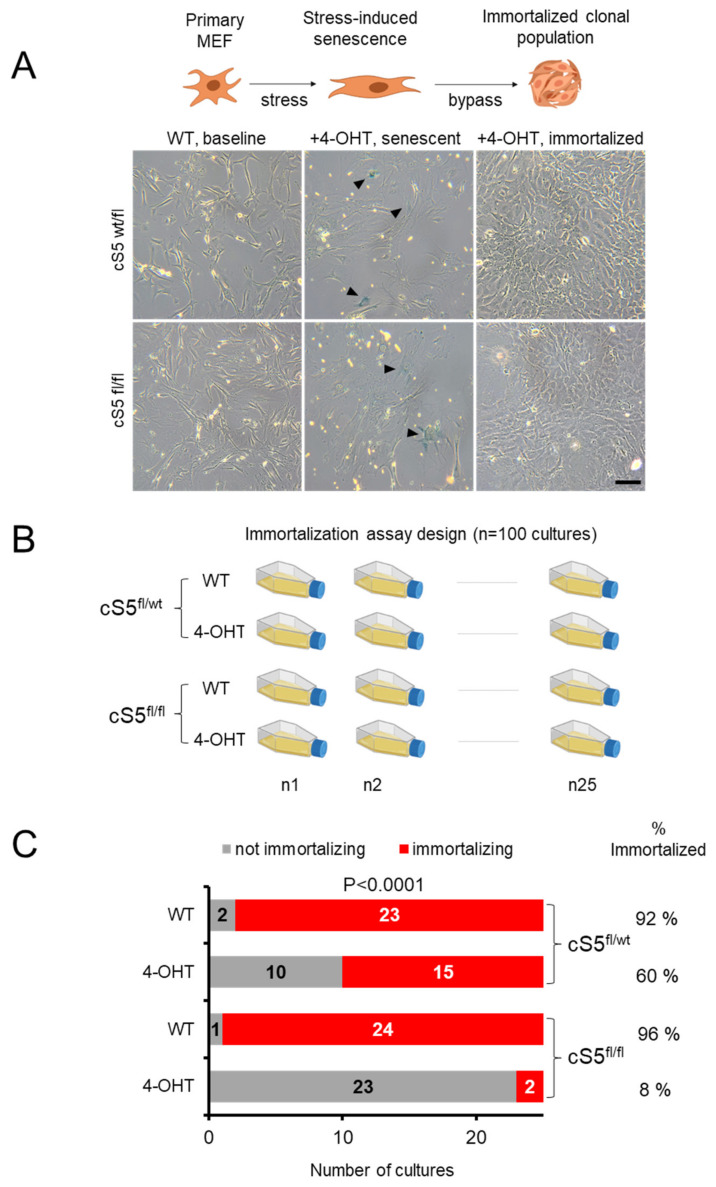
Large-scale analysis of the Smarca5 requirement for primary cell immortalization. (**A**) A schema showing senescence bypass and clonal immortalization in MEFs (top panel) and the representative cell culture images from the *Smarca5* single and double allele knockout (bottom panel). Black arrowheads in the middle panels indicate the SA-β-gal staining of cells with typical senescent morphology. Scale bar = 50 μm. (**B**) Immortalization setup for *Smarca5* knockout MEFs, twenty-five flasks for each condition were cultured over periods of several weeks until they bypassed senescence or died, to check the ability of the MEFs to overcome senescence and immortalize; created with BioRender.com. (**C**) Bar graphs show the total number of cultures out of twenty-five starting cultures that managed to immortalize when cells were grown for prolonged periods of time. NOTE: the *P*-value in (**C**) reflects the significance of difference between the conditions and it was calculated based on Χ^2^ test with the degree of freedom equal to 3.

We observed the dosage-dependent deregulation of numerous cancer driver genes, as well as of expression programs involving the gradual downregulation of proliferation and upregulation of apoptosis and cellular senescence, as an immediate- or intermediate–early response to *Smarca5* knockout induction in the primary MEFs. Based on this finding, we set out to determine the long-term phenotypic impact of Smarca5 loss on cellular fitness by taking advantage of the inherent ability of primary MEFs to bypass the prolonged SLS phase, induced by internal (e.g., oxidative/replicative) stress that can be accelerated by external stress factors (e.g., mutagen exposure), and to clonally immortalize (Figure 5A). To address this, we designed a large-scale functional experiment, involving 100 individual primary MEF cultures (Figure 5B). Twenty-five cultures each for the single and double allele knockout MEFs and the same number of corresponding un-induced wild-type cultures were grown for several months until they either bypassed senescence or died in the process. The loss of Smarca5 in MEFs resulted in accelerated growth arrest and senescent-like phenotype, compared to the wildtype controls (Figure 4 and Figure 5) [76]. Notably, the inherent ability of MEF cells to overcome senescence and immortalize was strongly compromised upon loss of Smarca5 in a dosage-dependent manner (Figure 5C). As expected, almost all wild-type cultures were immortalized. Single knockout cells overcame the SLS far less efficiently than wild-types and the double allele knockout MEFs rarely gave rise to immortalized cell lines. This experiment was repeated two more times applying the same general setup but using MEFs from different embryos and smaller numbers of individual cultures and resulted in the same findings (Supplementary Figure S11A). The *Smarca5* deletion status was confirmed by PCR and flow cytometric analysis after 4-OHT induction at the beginning of the experiment and then again by PCR in the immortalized knockout clones at the end of the experiment, to rule out the immortalization of rare wild-type cells within the *Smarca5* knockout population (data not shown). During the course of the experiment, we further noticed that the *Smarca5* knockout cultures that did overcome senescence took longer to do so than their corresponding wild-type controls (Supplementary Figure S11B). These findings, together with the reported molecular analyses, suggest an important role for Smarca5 in maintaining regulatory programs that favor cell proliferation, senescence bypass and primary cell immortalization, thereby contributing to the early stages of primary cell transformation. 

## 4. Discussion

We exploit an established mammalian model of primary cell immortalization to understand the role of an essential chromatin remodeler in the early stages of cell transformation using *omics* analyses and functional phenotypic validations. The differential gene expression changes observed are in keeping with previously observed roles of Smarca5 in cell cycle progression [24,30], initiation of DNA replication at origins [77] and the finding that the absence of Smarca5 inhibits replication fork velocity [78], thus retarding cell proliferation. Likewise, upregulation of the p53 pathway [79], including its downstream effector Cdkn1a, has been reported upon Smarca5 loss [30]. Altogether, we observe pronounced deregulation of genes contributing to overall cellular fitness, including downregulation of genes involved in cell proliferation, genomic stability and DNA damage repair, while senescence- and apoptosis-related genes were upregulated. Based on the stepwise reduction of *Smarca5* dosage, we were able to define a comprehensive set of gene expression and chromatin accessibility changes associated with Smarca5 loss, while minimizing the contribution of secondary alterations due to effects on cell health and viability observed in homozygous knockout cells. These molecular changes are supported by functional observations in *Smarca5* knockout MEFs: (i) the gradually increased sensitivity of knockout cells to exposure with chemical mutagens (wild-type < single-allele knockout < double-allele knockout), and (ii) the *Smarca5* dosage-dependent failure of primary MEFs to escape senescence and immortalize. These findings imply, for the first time, an important role for Smarca5 in cell immortalization, one of the critical initial steps during the transformation process. Interestingly, this is in agreement with the predominantly observed upregulation of *Smarca5* in human cancer [24,27,28,80,81]. Moreover, the deregulated expression of many cancer genes in our data reinforces the link between Smarca5 and the control of important cancer-related pathways.

Suppression of Smarca5 renders human cells sensitive to X-rays [75] and other studies reported similar results for ionizing radiation [21,36]. Interestingly, we recapitulate similar results with chemical mutagens in the exposure experiments, and because we could eliminate that differences in sensitivity to mutagen exposure stem from underlying genetic differences between the crosses, we propose that the sensitivity to versus tolerance of mutagen exposure is at least partially attributable to DNA damage repair defects. These observations are in concurrence with previous studies [74,75] showing a role of Smarca5 in DNA repair, and with the presented gene expression data showing downregulation of several pathways involved in repair in primary MEFs. SMARCA5 rapidly accumulates at DNA damage sites, where it is essential for the repair of DSBs [75,82] by recruitment of RAD51 and BRCA1 [33], and we observe downregulation of *Rad51* and *Brca1* gene expression as well as HR and NHEJ pathway genes in our experimental model. Moreover, interference with cohesin function has been shown to impede DNA repair [83,84] and several genes involved in sister chromatid cohesion are downregulated in our data. Chromosomal aberrations, polyploidy [34] and cohesion defects [85], all of which are linked to genomic stability, have been reported in the absence of Smarca5. We also observed the downregulation of genes involved in telomere maintenance. Together, these findings point towards an important role of Smarca5 for genomic stability/fitness in primary MEFs.

The deregulation of ERGs in the absence of Smarca5 raises the possibility that Smarca5 is not only directly involved in chromatin-associated functions through its role as an ATPase but also by affecting the transcription of other genes involved in these processes. As the absence of Smarca5 initiates a regulatory cascade involving the misexpression of other epigenetic regulator genes, the applied experimental setup does not allow a distinction between genes regulated by Smarca5 in a direct versus indirect manner. This is due to the general unavailability of well-performing Smarca5 antibodies for chromatin-immunoprecipitation and the current lack of publicly available good-quality data for Smarca5 ChIP-seq in MEFs. Future studies aimed to better understand the role of Smarca5 will strongly depend on the development of new experimental tools to circumvent these issues. 

## 5. Conclusions

The ability of primary cells to overcome cellular senescence and subsequent immortalized growth is a key characteristic of cancer formation. We describe the gene dosage-dependent compromised the ability of Smarca5-depleted MEFs to undergo these processes, in conjunction with deregulation of corresponding gene regulatory programs, thus directly implicating this important chromatin remodeler as a key determinant for cell fate decisions associated with early stages of cell transformation and initiation of cancer development. Follow-up characterization of direct contributions of Smarca5 to the observed gene regulatory programs, as well as the roles of other chromatin remodeling factors during primary cell immortalization, is warranted to better understand the maintenance of cellular and genomic health and the possible roles of chromatin remodelers in cancerous transformation.

## Data Availability

The original sequencing data is publicly available from the NCBI’s Sequence Read Archive (SRA) under the accession number PRJNA746698.

## Supplementary Materials

The following supporting information can be downloaded at https://www.mdpi.com/article/10.3390/cells11050808/s1, Figures S1–S11, Tables S1–S14.
