# Supplementary material for: Chromatin Remodeler Smarca5 Is Required for Cancer-Related Processes of Primary Cell Fitness and Immortalization
Source: Cells. 2022 Feb 25;11(5):808. doi: 10.3390/cells11050808 (PMC8909548; doi:10.3390/cells11050808)
Supplement: Supplementary file 1 [file cells-11-00808-s001.zip › Thakur_S_Supplementary_Figures_Smarca5_Cells_R2.pdf]

Suppl. Figure S1

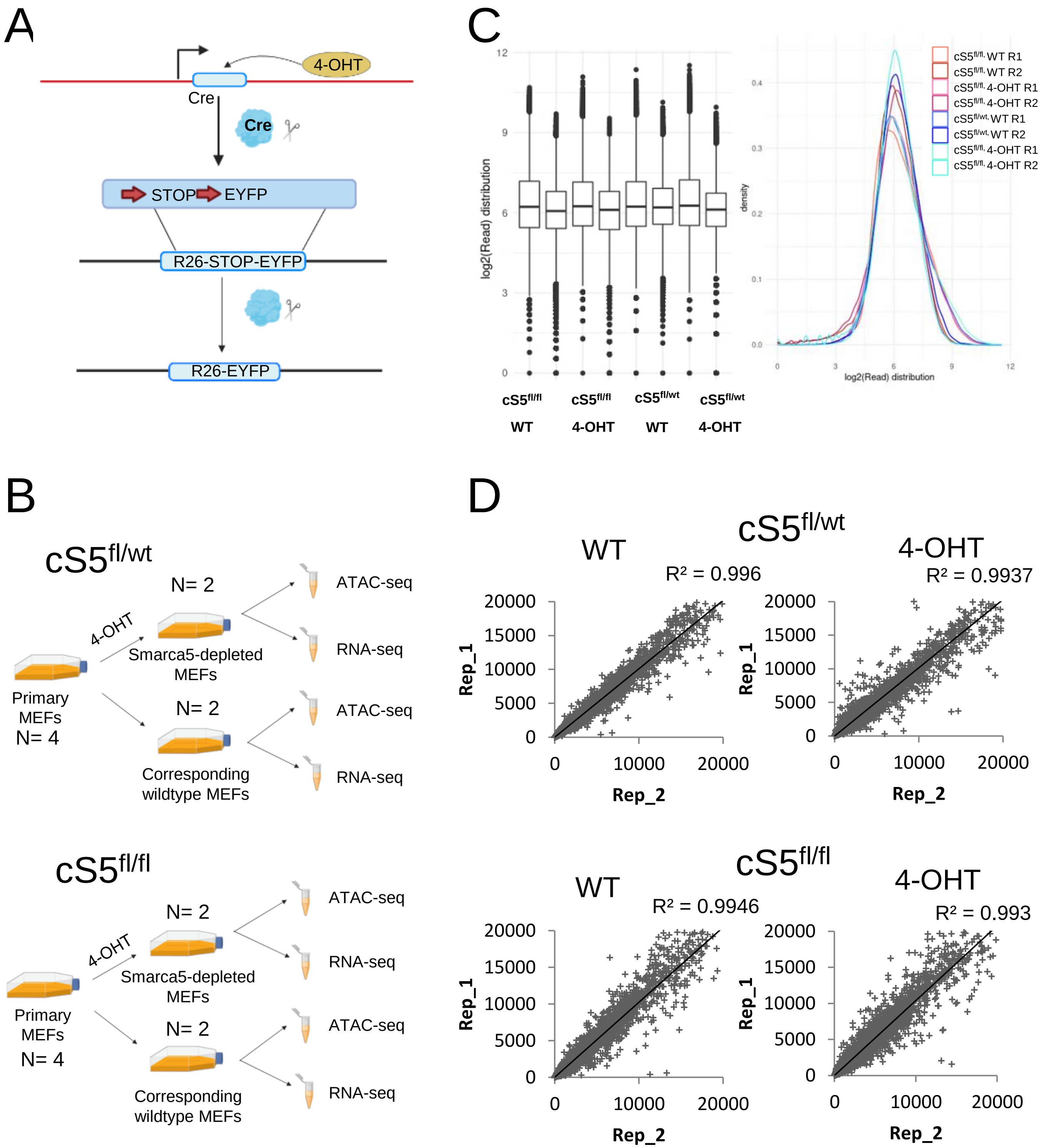

Supplementary Figure S1. A) Schematics of the conditionally expressed EYFP cassette. The *lox-P* flanked STOP sequence is excised upon 4-OHT induction and the EYFP is expressed in *Cre*-expressing cells of the double mutant MEFs. Image created with BioRender.com **B)** Study design for assessing chromatin accessibility and gene expression changes upon Smarca5 deletion in single allele (top) and double allele knockout (bottom) MEFs. The readouts are taken four days (96 hours) after the knockout. **C)** Box plot displaying log2 transformed normalized read distribution for ATAC-seq samples (left) and line plot displaying density of read distribution for the same samples (right). **D)** RNA-seq correlation plots for read counts between replicates.

Suppl. Figure S2

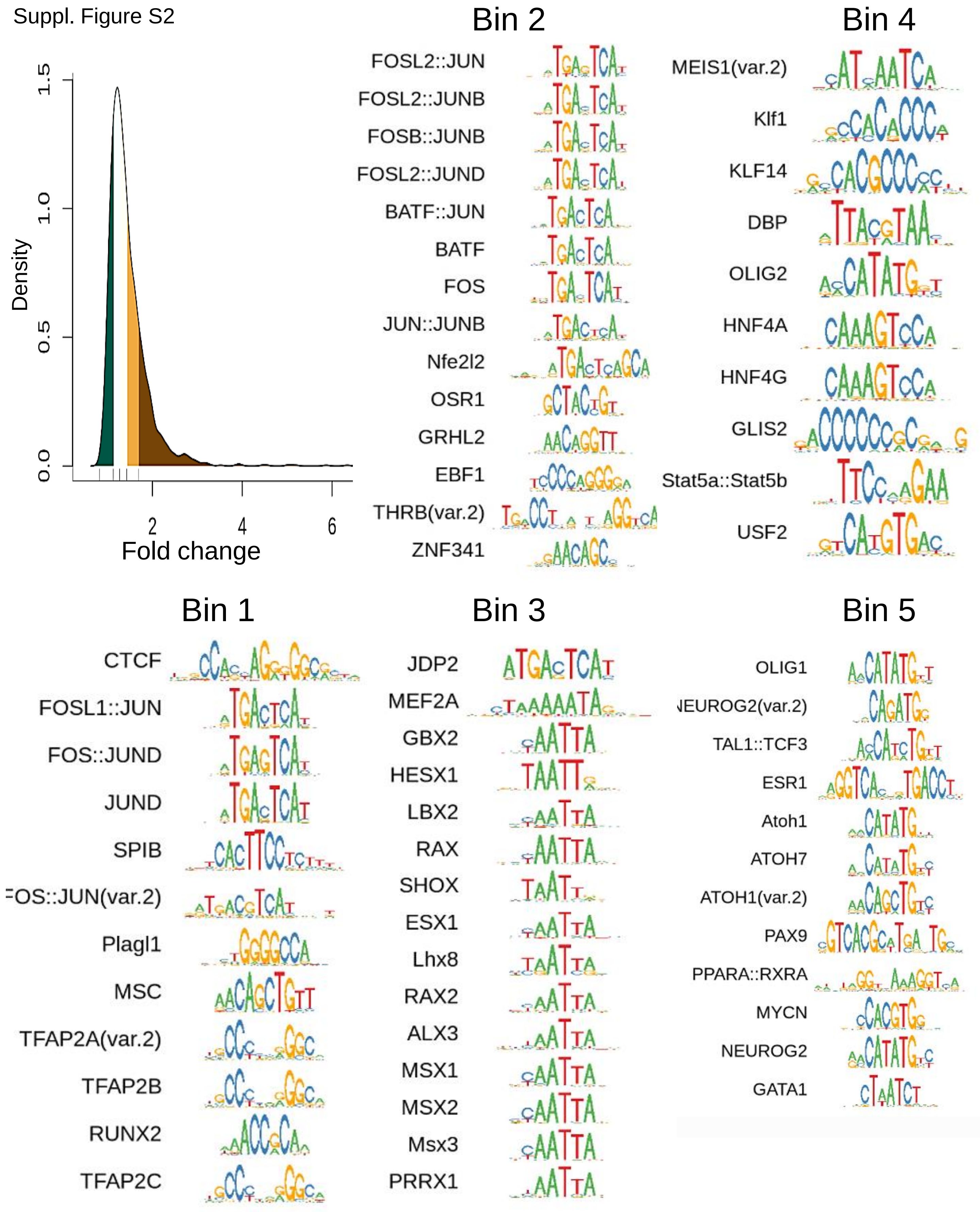

Supplementary Figure S2. Motif analysis of LASs in Smarca5 single-allele knockout MEFs as assessed by ATAC-seq. Motif analysis using HASs didn’t result in significant enrichment of motifs in any bin.

Suppl. Figure S3

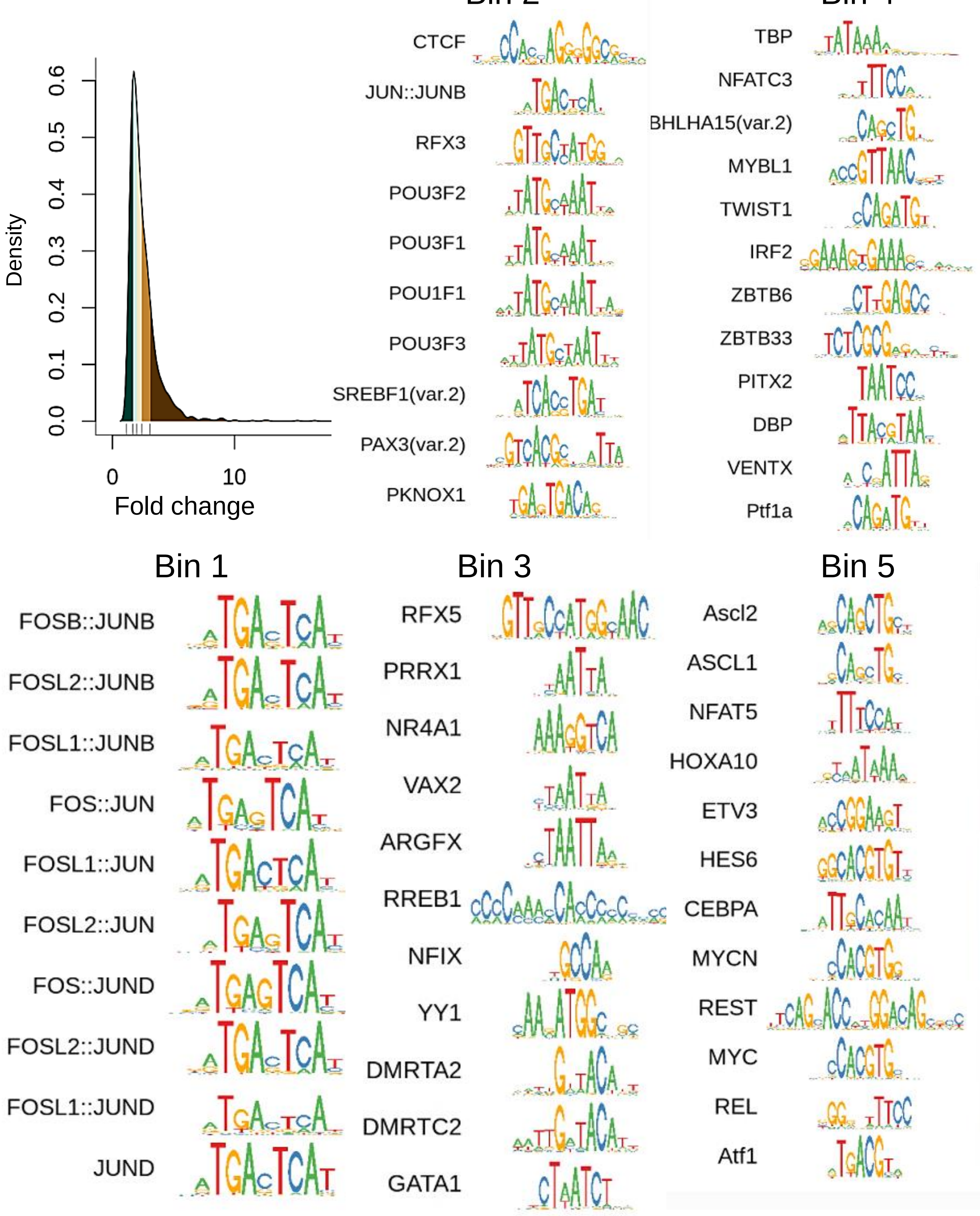

Supplementary Figure S3. Motif analysis of LASs in Smarca5 double allele knockout MEFs as assessed by ATAC-seq. Motif analysis using HASs didn’t result in significant enrichment of motifs in any bin.

Suppl. Figure S4

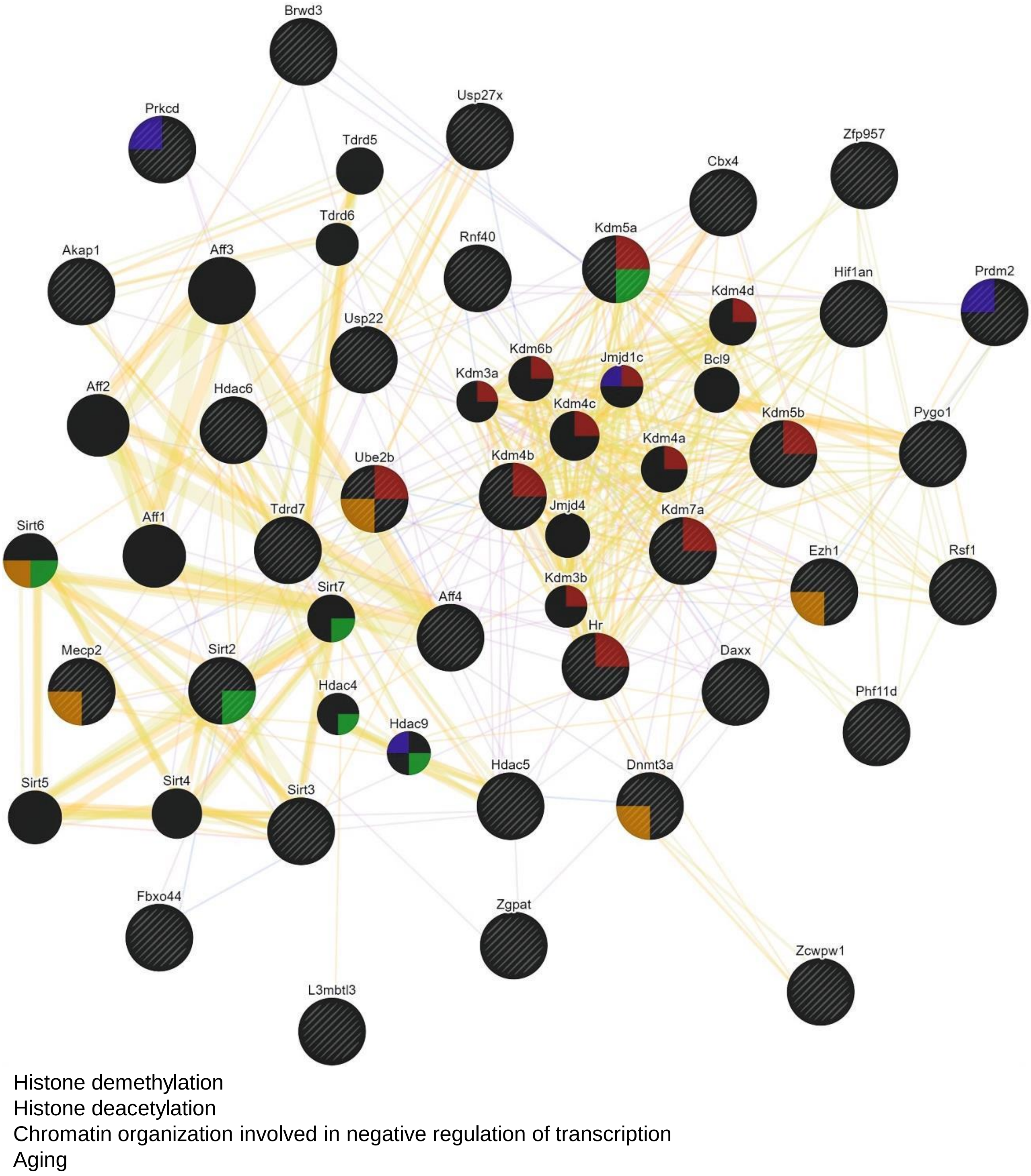

Supplementary Figure S4. GeneMANIA network map of gradually upregulated ERGs in Smarca5 knockout MEFs. Network lines represent GeneMANIA network categories – yellow: predicted, purple: co-expression, pink: physical interactions, blue: co-localization, green: genetic-interactions.

Suppl. Figure S5

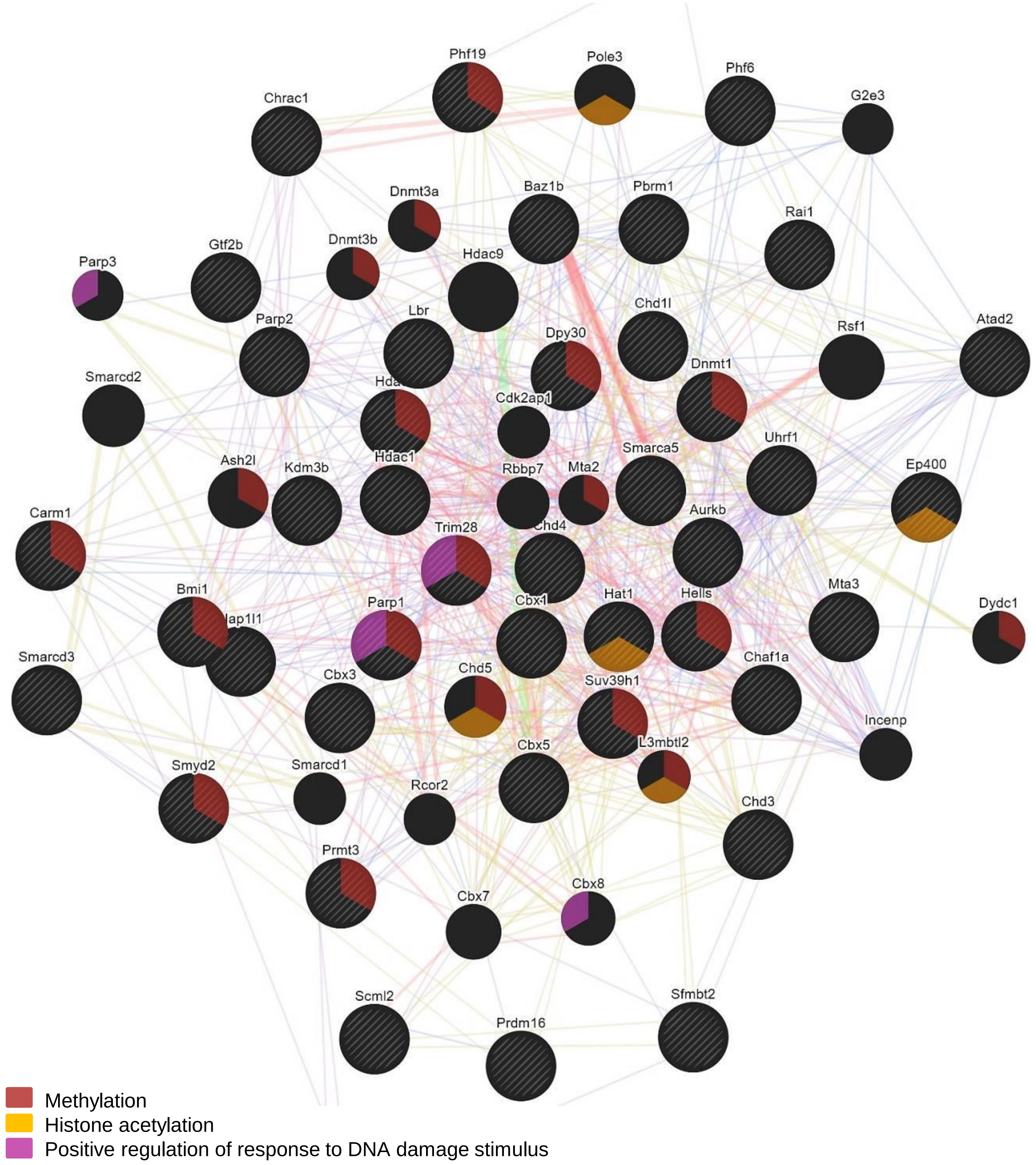

Supplementary Figure S5. GeneMANIA network map of gradually downregulated ERGs in Smarca5 knockout MEFs. Network lines represent GeneMANIA network categories – yellow: predicted, purple: co-expression, pink: physical interactions, blue: co-localization, green: genetic-interactions.

Suppl. Figure S6

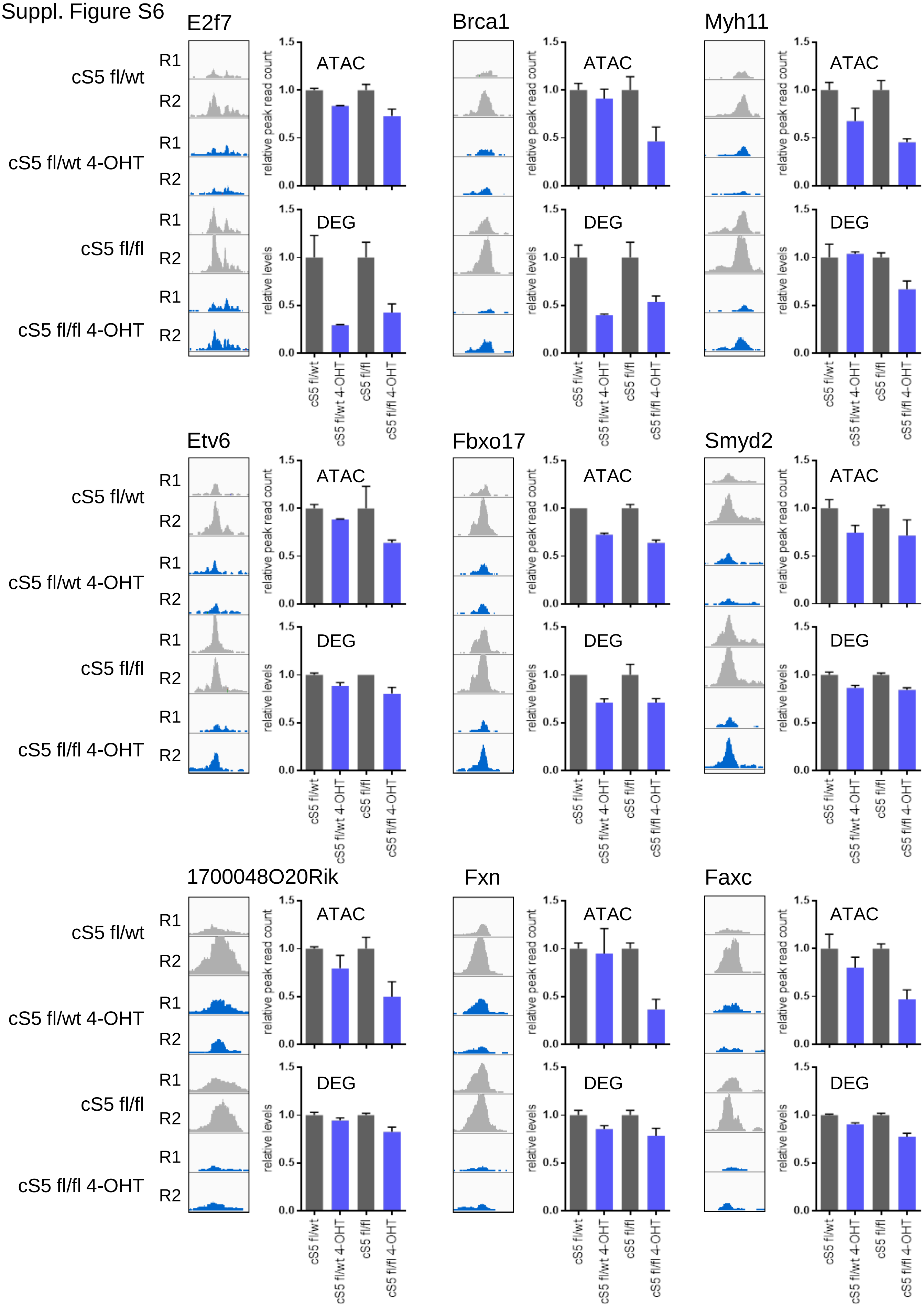

Supplementary Figure S6. Concordance of ATAC-seq and RNA-seq MEF cell data for decreased accessibility of select regulatory regions (low accessibility sites, LASs) and corresponding genes (DEGs) down-modulated upon stepwise knock-out of Smarca5. Vertical histograms display the ATAC-seq peaks for replicate (R1, R2) experiment across the studied genotypes (note that peaks represent *raw data, before across-experiment normalization* shown in Suppl. Fig. S1C). The adjacent bar graphs show normalized relative peak read counts for ATAC-seq (top graph) and the normalized relative mRNA levels (bottom graph) for a given gene. The top two rows show six enhancers and their regulated genes, the bottom row shows 3 proximal promoters and their genes. cS5 – conditional Smarca5 KO; fl/wt and fl/fl – single- and double allele knock-out, respectively; 4-OHT – treatment with 4-hydroxy-tamoxifen.

Suppl. Figure S7

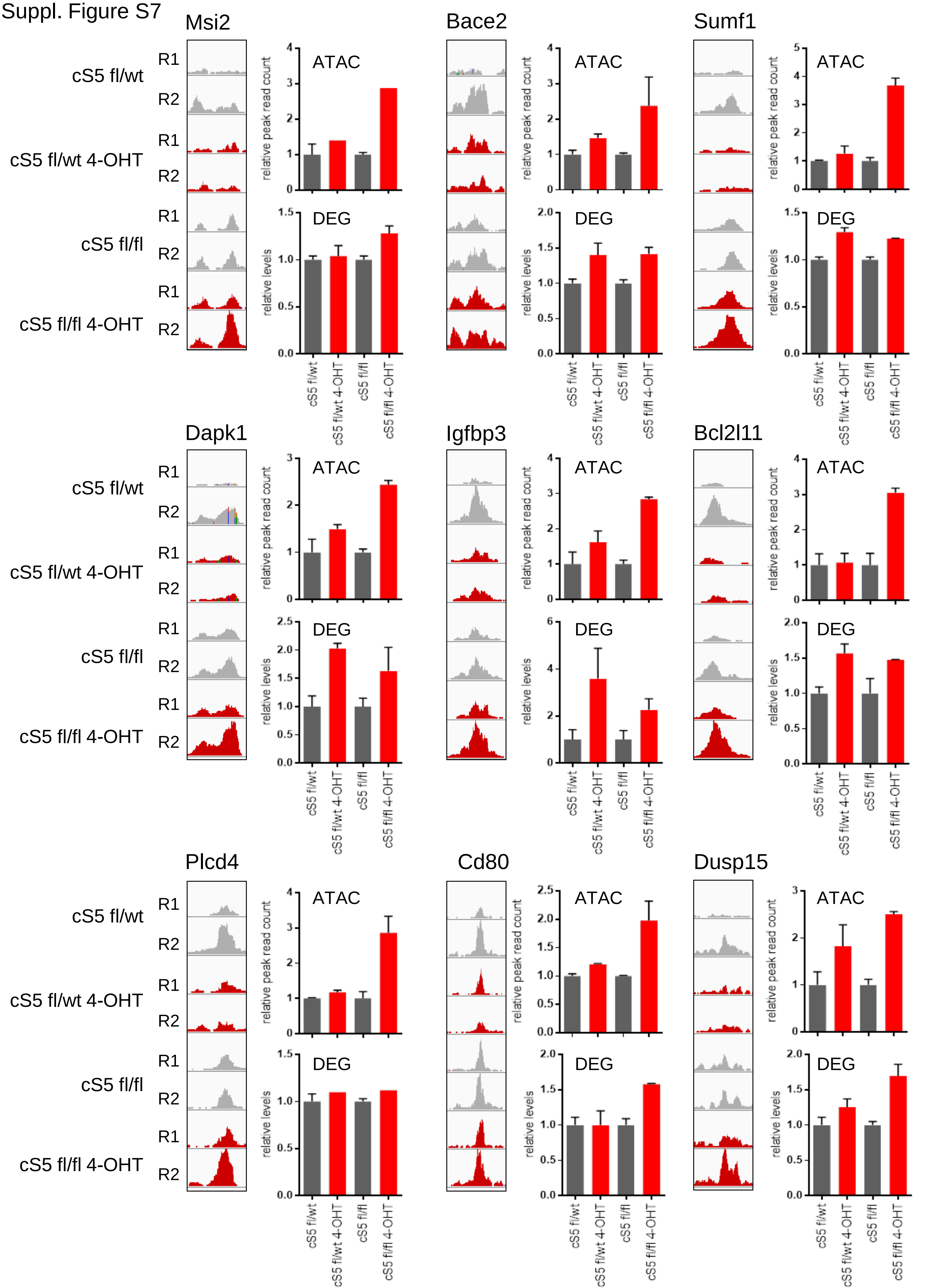

Supplementary Figure S7. Concordance of ATAC-seq and RNA-seq MEF cell data for increased accessibility of select regulatory regions (high accessibility sites, HASs) and corresponding genes (DEGs) up-modulated upon stepwise knock-out of Smarca5. Vertical histograms display the ATAC-seq peaks for replicate (R1, R2) experiment across the studied genotypes (note that peaks represent *raw data, before across-experiment normalization* shown in Suppl. Fig. S1C). The adjacent bar graphs show normalized relative peak read counts for ATAC-seq (top graph) and the normalized relative mRNA levels (bottom graph) for a given gene. The top two rows show six enhancers and their regulated genes, the bottom row shows 3 proximal promoters and their genes. cS5 – conditional Smarca5 KO; fl/wt and fl/fl – single- and double allele knock-out, respectively; 4-OHT – treatment with 4-hydroxy-tamoxifen.

Suppl. Figure S8

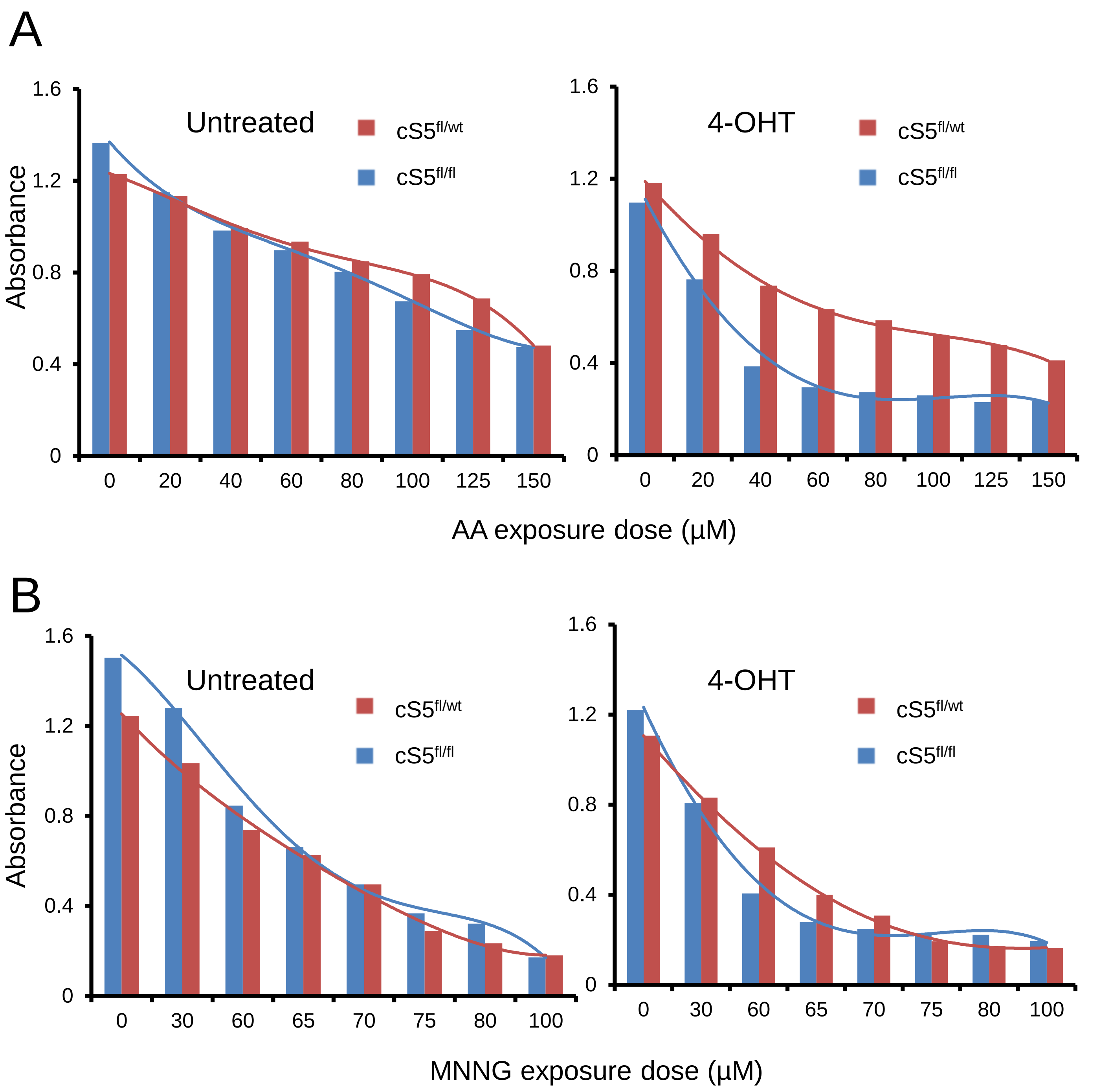

Supplementary Figure S8: A) Comparison of the proliferative differences as assessed by absolute absorbance measure via MTS assay of single and double allele knockout (right) and their respective wildtypes (left) side-by-side upon exposure to various doses of aristolochic acid-I (AA). **B)** Comparison of the proliferative differences as assessed by absolute absorbance measure via MTS assay of single and double allele knockout (left) and their respective wildtypes (right) side-by-side upon exposure to various doses of methylnitronitrosoguanidine (MNNG).

Suppl. Figure S9

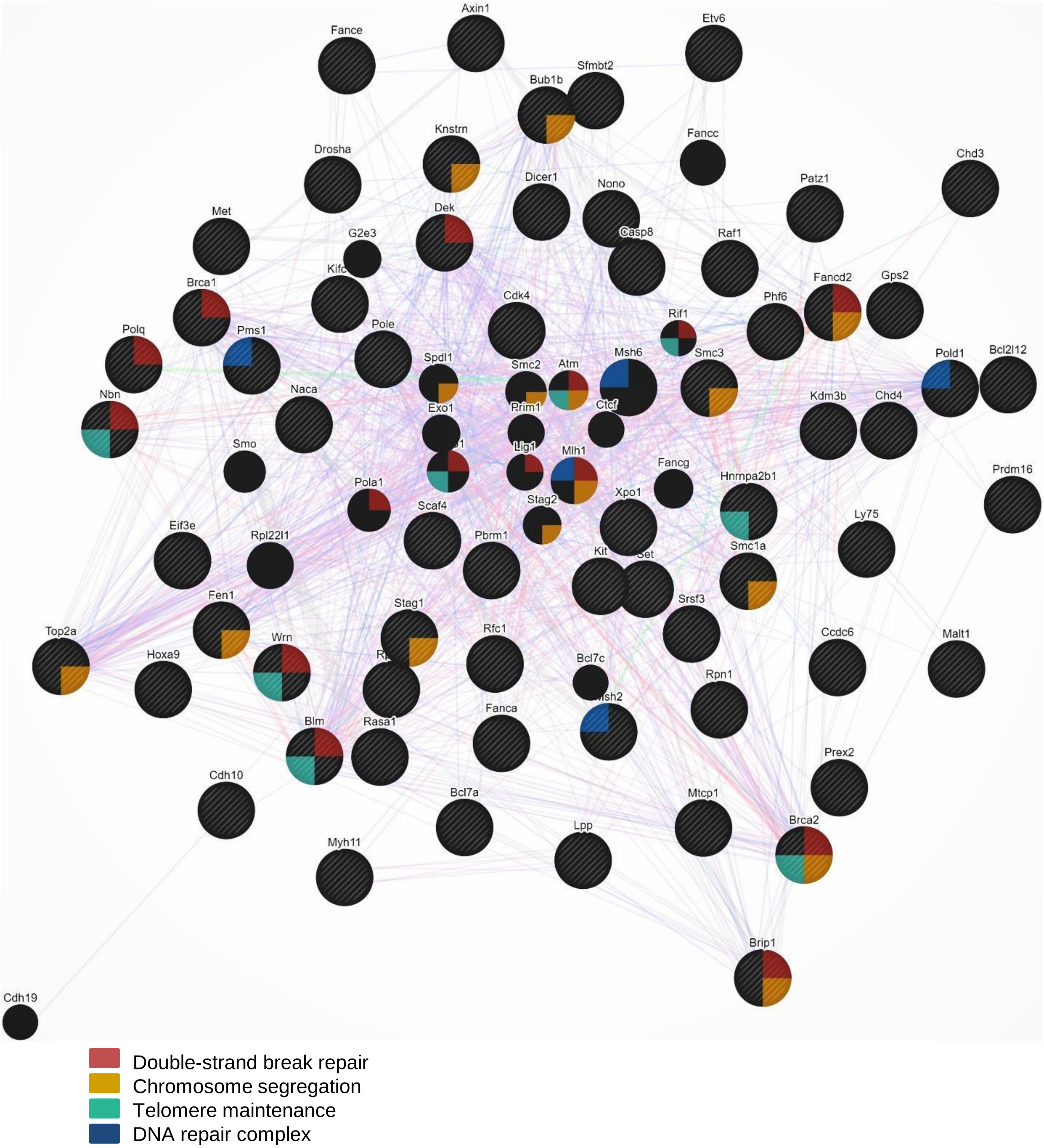

Supplementary Figure S9. The interaction network map of gradually downregulated cancer driver genes in Smarca5 knockout MEFs. Network lines represent GeneMANIA network categories – purple: co-expression, pink: physical interactions, blue: co-localization, green: genetic-interactions.

Suppl. Figure S10

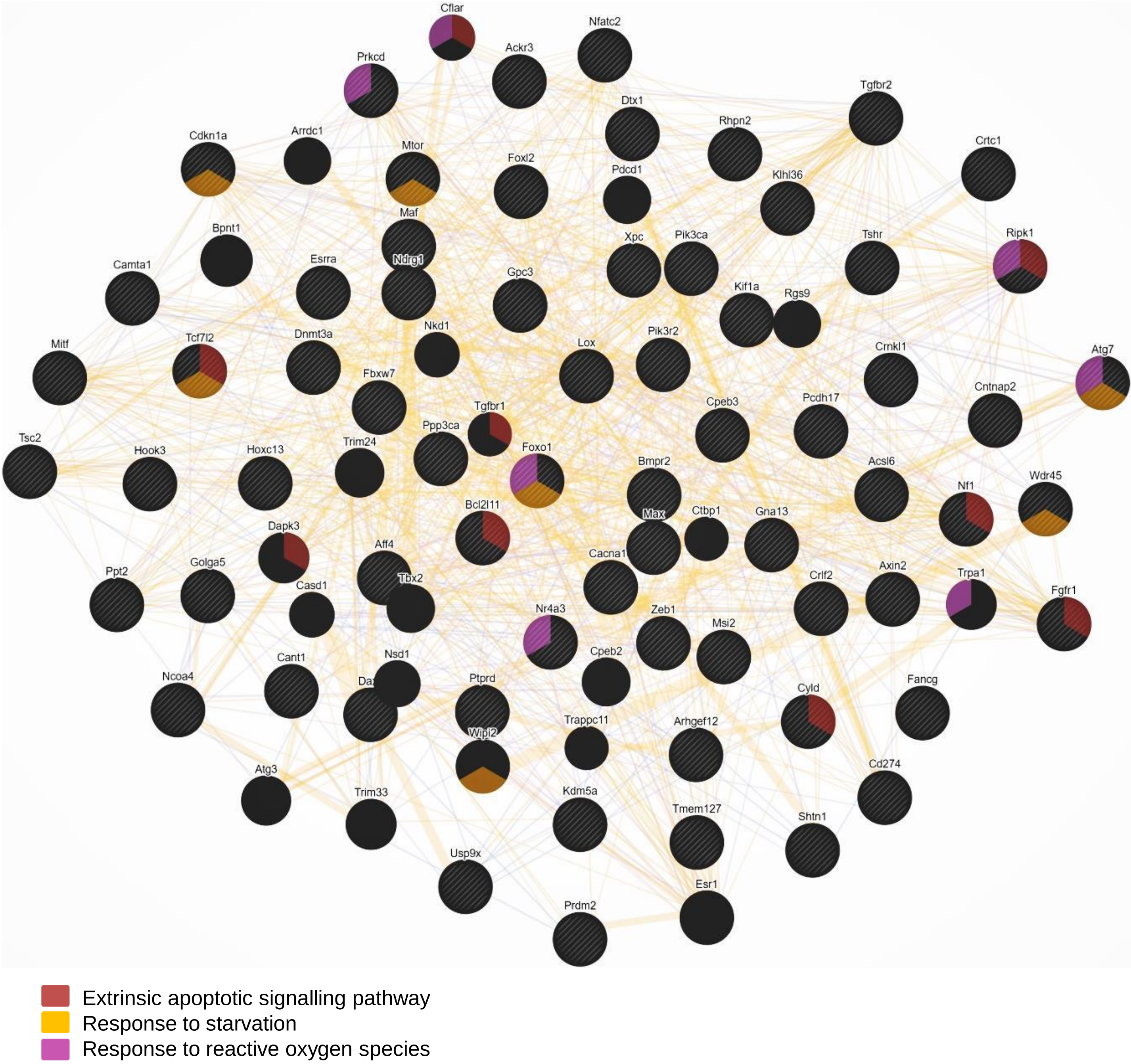

Supplementary Figure S10. GeneMANIA network map of gradually upregulated cancer driver genes in Smraca5 knockout MEFs. Network lines represent GeneMANIA network categories – yellow: predicted, purple : co-expression, pink : physical interactions, blue : co-localization, green : genetic-interactions.

Suppl. Figure S11

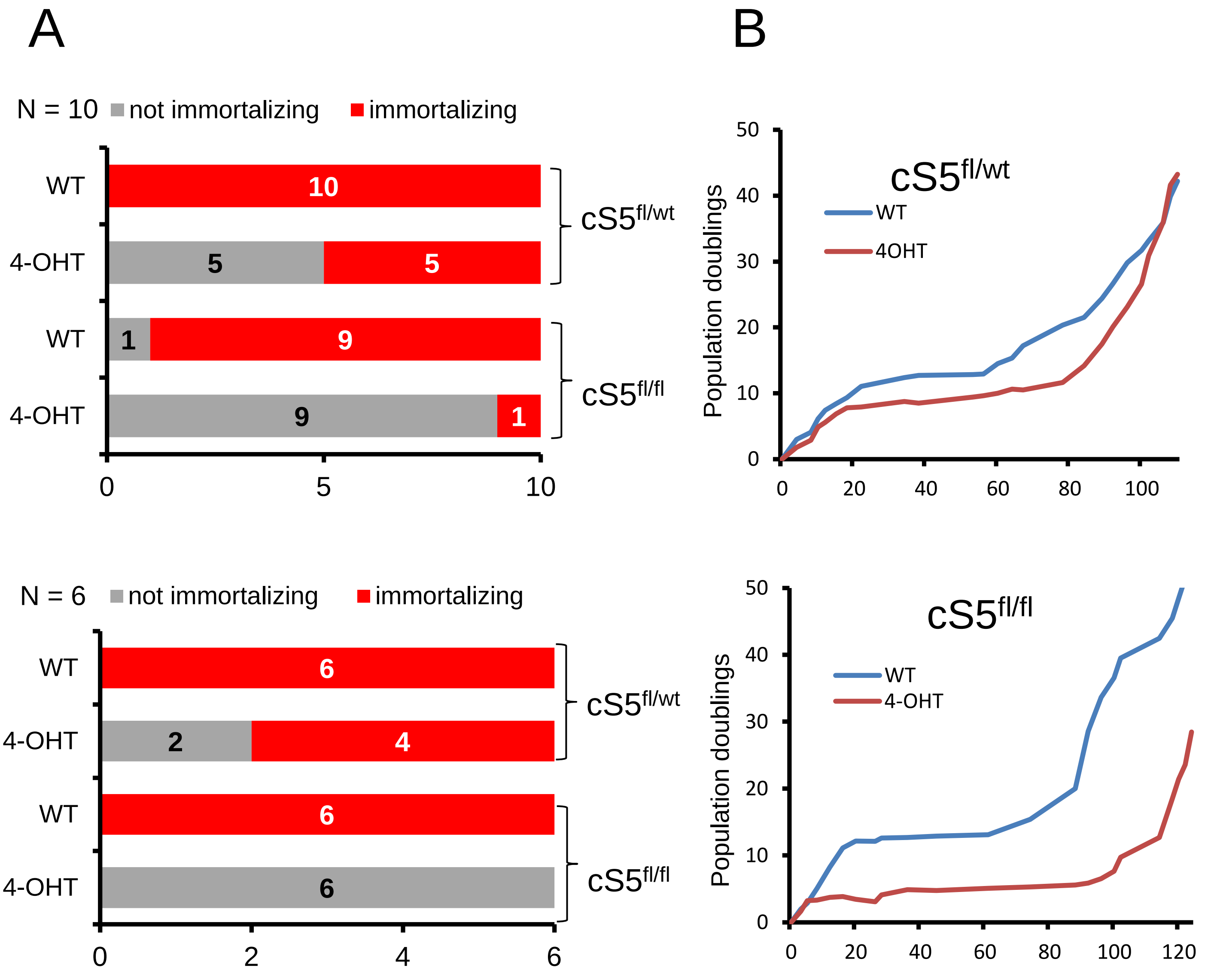

Supplementary Figure S11. A) Bar graphs show the total number of cultures out of ten (top) and six (bottom) starting cultures that managed to immortalize when cells were grown for prolonged periods of time, WT = untreated with 4-OHT. **B)** Growth curves of the single allele (top) and double allele (bottom) knockout MEFs with their corresponding wildtypes that bypassed senescence and immortalized.
